# Shift in Patient Demographics of Open Thoracoabdominal Aortic Aneurysm Repair Patients in the Endovascular Era

**DOI:** 10.3390/jcm14197088

**Published:** 2025-10-08

**Authors:** Jelle Frankort, Siebe Frankort, Panagiotis Doukas, Christian Uhl, Moustafa Elfeky, Barend M. E. Mees, Alexander Gombert, Michael J. Jacobs

**Affiliations:** 1 Department of Vascular Surgery, Medical Faculty, RWTH Aachen University, 52074 Aachen, Germany; 2 Department of Vascular Surgery, MUMC+ Maastricht, 6229 HX Maastricht, The Netherlands; 3 Institute of Statistics Netherlands (CBS), 6401 CZ Heerlen, The Netherlands

**Keywords:** thoracoabdominal aortic aneurysms, open surgical repair, patient demographics, indications

## Abstract

**Background/Objectives**: Open thoracoabdominal aortic aneurysm (TAAA) repair remains essential despite expanded endovascular options, yet the contemporary open-surgery case-mix has shifted as minimally invasive therapies became widespread. The objective was to evaluate temporal changes in patient demographics, pathology, and perioperative outcomes of open TAAA repair across two decades. **Methods**: Retrospective, cross border cohort of all open TAAA repairs performed at two high-volume tertiary centers (Aachen, Germany; Maastricht, Netherlands) from 2000–2024. Patients were stratified into Early Era (2004–2013) and Late Era (2014–2024). Primary endpoints were shifts in demographics and perioperative mortality/morbidity; secondary endpoints included major complications (spinal cord ischemia, acute kidney injury, pulmonary and cardiac events). **Results**: Among 577 open repairs, 376 (65.2%) occurred in the Early Era and 201 (34.8%) in the Late Era, with annual volumes declining to <12 cases/year after 2020. Late Era patients were younger (median 55.9 vs. 63.0 years, p<0.001) and had more genetic aortopathy (Marfan 26.9% vs. 11.7%, p<0.01) and post-dissection pathology (64.7% vs. 43.1%, p<0.01), alongside more prior aortic surgery (59.2% vs. 43.4%, p<0.01). Massive transfusion and incidental splenectomy decreased (37.8% vs. 54.5%, p<0.01; 5.0% vs. 14.9%, p<0.01). In-hospital mortality was similar (18.4% Late vs. 21.8% Early, p=0.34); spinal cord ischemia showed a non-significant reduction (5.5% vs. 8.0%, p=0.26); myocardial infarction decreased (1.0% vs. 4.3%, p=0.03); and ARDS increased (15.9% vs. 5.1%, p<0.01). **Conclusions**: Despite the shift towards endovascular repair and the changing demographics of patients selected for open TAAA repair, specialized centers can maintain stable outcomes through standardized protocols and concentrated expertise. The preservation of open surgical capabilities remains crucial for specific patient populations, emphasizing the need for a balanced approach that integrates both open and endovascular techniques to provide optimal, individualized care.

## 1. Introduction

Thoracoabdominal aortic aneurysms are uncommon but high-risk pathologies in which repair remains technically demanding and resource-intensive despite advances in perioperative care and organ-protection strategies [1,2]. For decades, open repair remained the cornerstone of management. Despite advancements in perioperative care, spinal cord protection protocols, and graft design, the procedure has been related to significant morbidity, including risks of spinal cord ischemia, renal failure, and mortality, which have driven efforts to refine patient selection and optimize outcomes [3,4].

The TAAA management underwent a shift following the introduction and progressive refinement of endovascular techniques. The year 2014 marks a change in the management of TAAAs, as evidenced by Geisbüsch et al. analysing nationwide German data from 2005 to 2014 [5]. Their findings reveal a relevant increase in endovascular procedures for TAAA repair, with the proportion rising from just 6% in 2005 to 76% in 2014 for non-ruptured TAAAs. Since 2014, the endovascular treatment option has precipitated a marked decline in open repair volumes. This transition has not only expanded treatment options but also fundamentally altered the clinical and anatomical profile of patients referred for open surgery. Prior to the endovascular era, open repair was broadly applied across diverse patient cohorts, with a wide spectrum of aneurysm morphologies and risk profiles [1,2].

These changes raise practical questions that are insufficiently characterized in studies: how the open case-mix has evolved within specialized centers; whether perioperative outcomes have been maintained amid shifting indications; and which patient or perioperative factors independently drive mortality in current practice. Early reports indicate a trend toward younger age at intervention, increased prevalence of genetically triggered aortopathies, and a focus on aneurysms with anatomical complexity less amenable to endovascular solutions [6,7,8,9].

This study characterizes how case-mix has evolved across two eras and evaluates perioperative outcomes and independent mortality predictors in a contemporary open TAAA cohort.

## 2. Materials and Methods

### 2.1. Study Design and Setting

This retrospective, cross-border cohort study analysed all TAAA repairs performed between January 2000 and January 2024 at two high-volume academic vascular centers: University Hospital RWTH Aachen, Germany, and Maastricht University Medical Center, Netherlands. Both hospitals are tertiary care referral centers with identical surgical and neuromonitoring protocols in both locations.

### 2.2. Patient Selection and Cohort Stratification

Patients were included if they underwent open surgical repair for TAAA during the study period. TAAAs were classified according to the Crawford and Safi classification (types I–V) [10]. Both elective and emergency cases were included. Patients were divided into two cohorts: Early Era (2004–2013) and Late Era (2014–2024).

The primary outcomes were changes in patient demographics and perioperative mortality/morbidity between the pre-2014 and post-2014 cohorts. Secondary outcomes included major postoperative complications such as spinal cord ischemia, acute kidney injury, and reintervention rates.

### 2.3. Surgical Protocol

The surgical protocol employed at our institution during the study period has been previously described in detail [3]. Operative technique, adjuncts, and perioperative pathways were standardized between centers and remained largely unchanged over the study period. The surgical approach involved selective double-lumen endotracheal intubation combined with cerebrospinal fluid drainage and continuous perioperative monitoring of motor-evoked potentials. Patients were placed on a beanbag in a modified right lateral decubitus position, with the operating table extended to optimize exposure of the thoracic cavity. The procedure incorporated sequential aortic clamping, femoro-femoral extracorporeal circulation with distal aortic perfusion, selective visceral perfusion, and induction of mild systemic hypothermia (32–33 °C). The choice of incision and specific technical modifications were adapted according to the extent of the aneurysm [3].

### 2.4. Definitions

Emergency repair was considered any intervention performed within 24 h due to symptomatic or ruptured TAAA. Chronic kidney disease was defined as a preoperative estimated glomerular filtration rate (eGFR) below 60 mL/min/1.73 m^2^. AKI occurring within 48 h after surgery was classified according to the Kidney Disease: Improving Global Outcomes (KDIGO) criteria based on serum creatinine changes [10]. Operative mortality included all deaths within 30 days of the procedure or during the index hospitalization. Pulmonary complications comprised pneumonia, prolonged mechanical ventilation requiring tracheostomy, and acute respiratory distress syndrome. Reoperation referred to any surgical intervention after initial hospital admission. Sepsis and shock were defined in accordance with current clinical guidelines [11]. Cardiac complications included myocardial infarction, acute heart failure, and ventricular tachycardia. Massive transfusion was defined as the intraoperative administration of 10 or more units of packed red blood cells.

### 2.5. Statistical Analysis

Statistical analyses were conducted using RStudio (Version number: 2024.04.0, R Core Team, PBC, Boston, MA, USA) with packages obtained from the CRAN repository. Continuous variables are reported as medians with corresponding minimum and maximum values, while categorical variables are expressed as absolute numbers (n) and percentages. Comparisons were performed for those treated before 31 December 2013, and those treated thereafter. Categorical variables were analyzed using the Chi-squared test, whereas continuous variables were compared using the Mann–Whitney U test. A *p*-value of <0.05 was considered statistically significant. To identify independent predictors of operative mortality (defined as in-hospital or 30-day mortality), multivariable logistic regression models were developed based on clinically relevant variables selected a priori.

### 2.6. Ethical Considerations

This study, approved by the local internal review board (EK004/14, approval date 20 April 2020), was reported according to the STROBE criteria [12]. Following protocol approval, clinical data for patients undergoing open TAAA repair were collected prospectively. Following approval, written informed consent for all patients was achieved. For patients who were unable to provide consent due to the severity of their illness and who had no available family members to provide surrogate consent, a waiver of consent was granted by the institutional review boards. For the period prior to protocol approval, data were collected retrospectively from medical records. For this period, a waiver of consent to collect retrospective data was also approved by the institutional review boards. Retrospective data collection started in 2020 and was collected by experienced researchers. Standardized case-report forms and dual verification of key endpoints were used across both phases to ensure data quality.

## 3. Results

**Figure 1:**
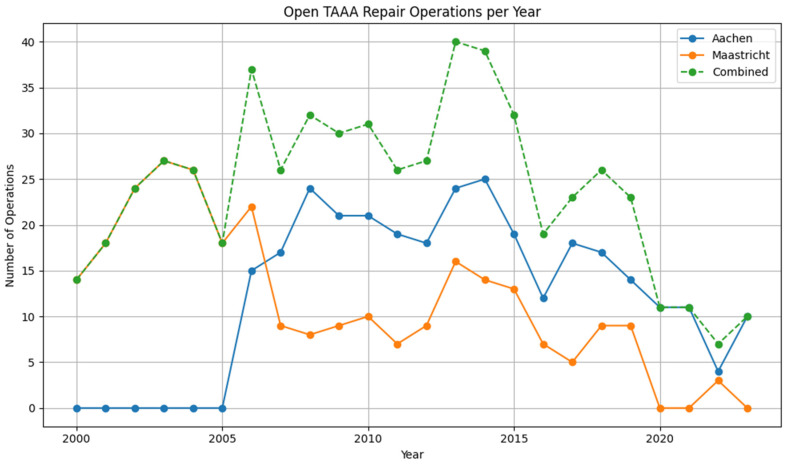
Annual count of open TAAA repair operations at University Hospital Aachen, Maastricht University Medical Center, and the combined total.

During the study period from 2000 to 2024, a total of 577 patients underwent open thoracoabdominal aortic aneurysm (TAAA) repair across both participating centers. The cohort was divided into two distinct eras: 376 patients (65.2%) were treated in the Early Era (2004–2013), while 201 patients (34.8%) underwent repair in the Late Era (2014–2024). The annual number of open TAAA repairs performed over the study period increased from the early 2000s, reaching a maximum of approximately 40 cases in 2013–2014. After this peak, the number of open repairs declined sharply, with fewer than 12 cases per year recorded from 2020 onwards. (Figure 1).

Patients in the Late Era were younger, with a median age of 55.87 years compared to 63 years in the Early Era (*p* < 0.001). This age difference represents a 7-year reduction in median age at the time of open repair. The prevalence of genetic aortopathy increased, particularly Marfan syndrome. In the Late Era, 54 patients (26.87%) suffered from Marfan syndrome compared to 44 patients (11.70%) in the Early Era (*p* < 0.001, OR 0.36, 95% CI 0.23–0.57) (Table 1).

Gender distribution remained stable between eras, with males comprising 68.35% of the Early Era cohort and 71.64% of the Late Era cohort (*p* = 0.41). BMI showed no significant temporal variation, with median values of 25.18 and 25.15 kg/m^2^ for Early and Late Eras, respectively.

Significant changes were observed in comorbidity patterns. ASA score distribution demonstrated a significant shift between the two eras. In the Early Era, 193 patients (51.33%) had ASA scores < 3, while 147 patients (39.10%) had ASA scores ≥ 3. In contrast, the Late Era showed a marked reversal with only 8 patients (3.98%) having ASA scores < 3 and 168 patients (83.58%) having ASA scores ≥ 3 (*p* < 0.001, OR 0.13, 95% CI 0.08–0.19). Patients in the Late Era demonstrated a lower prevalence of atherosclerotic risk factors: Previous myocardial infarction was substantially less common in the Late Era (8.46% vs. 35.37%, *p* < 0.001, OR 5.87, 95% CI 3.50–10.41). Although, heart failure was more prevalent in the Late Era (23.38% vs. 11.17%, *p* < 0.001).

Aortic dissection as the underlying pathology was significantly more common in the Late Era, affecting 130 patients (64.68%) compared to 162 patients (43.09%) in the Early Era (*p* < 0.001, OR 0.41, 95% CI 0.29–0.59). Additionally, patients in the Late Era were more likely to have undergone previous aortic surgery (59.20% vs. 43.35%, *p* < 0.001).

The number of urgency or emergency repairs remained relatively consistent between eras, with elective procedures comprising 80.85% and 85.07% of cases in the Early and Late Eras, respectively (*p* = 0.21).

Crawford classification distribution showed minimal variation between eras, with Crawford extent I aneurysms representing the most common extent in both periods (28.99% Early Era vs. 30.85% Late Era). Crawford extent V aneurysms showed a trend toward increased frequency in the Late Era (7.46% vs. 3.72%, *p* = 0.05).

Massive transfusion requirements decreased substantially from 54.52% in the Early Era to 37.81% in the Late Era (*p* < 0.001, OR 1.97, 95% CI 1.39–2.80). Incidental splenectomy was also significantly reduced in the Late Era (4.98% vs. 14.89%, *p* < 0.001, OR 3.30, 95% CI 1.71–7.04).

Operative times and hospital length of stay showed no significant differences between eras, with median surgery duration of 372.5 min in the Early Era versus 397 min in the Late Era (*p* = 0.07). (Table 2).

Overall pulmonary complications remained stable (59.04% Early Era vs. 60.20% Late Era, *p* = 0.79). However, acute respiratory distress syndrome (ARDS) was significantly more common in the Late Era (15.92% vs. 5.05%, *p* < 0.001, OR 0.28, 95% CI 0.15–0.51) (Table 3).

Overall cardiac complications were similar between eras (33.24% vs. 29.35%, *p* = 0.34), myocardial infarction was significantly less frequent in the Late Era (1.00% vs. 4.26%, *p* = 0.03, OR 4.14, 95% CI 1.15–28.64).

Acute kidney injury patterns differed between eras, with stage 2 AKI being more common in the Late Era (28.36% vs. 17.29%, *p* = 0.002, OR 0.53, 95% CI 0.35–0.80), stage 3 AKI also showed increased frequency (2.99% vs. 0.53%, *p* = 0.02).

Neurological complications, including spinal cord ischemia, remained comparable between eras. Spinal cord ischemia occurred in 7.98% of Early Era patients versus 5.47% of Late Era patients (*p* = 0.26), representing a non-significant trend toward reduced likelihood after 2014.

In-hospital mortality was 21.81% in the Early Era compared to 18.41% in the Late Era (*p* = 0.34, OR 1.23, 95% CI 0.80–1.92) (Table 3).

A breakdown per Crawford extent can be found in the Supplementary Material. (Supplementary Tables S1–S3).

### Multivariable Logistic Regression

In the full cohort, age (*p* = 0.01) and postoperative complications ARDS (*p* = 0.01) and AKI stage 2 and stage 3 (*p* = 0.02 and *p* = 0.03) were independently associated with higher operative mortality. In era-stratified analyses, massive transfusion (*p* = 0.02) was significant in the Early Era, whereas ARDS (*p* = 0.02) and incidental splenectomy (*p* = 0.04) were significant in the Late Era. The models for the logistic regression can be found in the Supplementary Material. (Supplementary Tables S4–S6).

## 4. Discussion

Our study demonstrates that despite certain shifts in patient demographics between the Early Era (2004–2013) and Late Era (2014–2024), mortality and complications following open thoracoabdominal aortic aneurysm repair have remained comparable. The combined in-hospital mortality rate was 21.81% in the Early Era compared to 18.41% in the Late Era (*p* = 0.34), indicating maintained surgical outcomes despite treating a substantially different patient population [13]. This preservation of outcomes is particularly noteworthy given the demographic transformation observed, including a significantly younger patient cohort (median age 55.87 vs. 63 years, *p* < 0.001) and a more than twofold increase in genetic aortopathies, particularly Marfan syndrome (26.87% vs. 11.70%, *p* < 0.001).

Our outcomes should be interpreted within the broader international landscape of open TAAA repair in the endovascular era. Recent multicenter data demonstrate considerable variation in operative mortality across specialized centers, with rates ranging from 4.9% to 20.6% for mixed elective-urgent cohorts. A large study of 2884 elective open TAAA repairs (1986–2023) from a leading US center reported operative mortality ranging from 3.6% to 15.9% depending on patient risk factors and extent [14]. In comparison, our combined 30-day mortality of 20.1% reflects the inclusion of urgent/emergency cases (17.7% of total). When stratified by era, our findings of stable mortality (21.8% Early vs. 18.4% Late Era, *p* = 0.34) despite significant demographic shifts mirror the experience of other European centers adapting to endovascular case selection [15]. Asian centers also show mixed results, where contemporary series report mortality rates ranging from less than 5% to over 20%, with the variation largely attributable to patient selection and institutional expertise [16,17]. A Korean high-volume center reported excellent outcomes with 5-year survival estimates of 94–95% in their recent decade experience, emphasizing the importance of specialized centers in achieving optimal results. The consistency of age, extent, and comorbidity burden as independent predictors across international cohorts validates our multivariable findings and underscores the importance of standardized risk assessment [14,17].

Interestingly, the rate of ARDS and AKI even increased in the late era. This may be attributable to the rising proportion of patients with genetic aortopathies and post-dissection pathologies which in turn leads to more complex aortic pathologies and technically more challenging repairs. Since patients with a genetic aortopathy have a higher rate of post-dissection TAAA and more often have previous aortic operations [18]. As well as patients with a genetic aortopathy often have underlying lung involvement or differences in chest wall mechanics [19,20]. In line with this, we observed that ASA score drastically increased and surgery time, although not significant, also increased in the Late Era. This in turn could also explain the higher rates of AKI. Since ASA score is a risk factor in cardiac surgery [21]. Favorable declines in massive transfusion and incidental splenectomy are best explained by cumulative technical and perioperative improvements rather than case simplification. Bleeding-related endpoints improved—consistent with refined aortic exposure, organ-sparing maneuvers, targeted hemostasis, and blood-conservation protocols. In multivariable analyses, massive transfusion was a significant mortality predictor in the Early Era but not in the Late Era.

The most striking temporal change observed in our study was the decline in open repair volume following 2014, this correlates directly with the nationwide German experience documented by Geisbüsch et al., where endovascular procedures increased from 6% in 2005 to 76% in 2014 for non-ruptured TAAAs [5]. The volume reduction observed in our centers represents more than a 70% decrease from peak levels, consistent with reports from other major aortic centers worldwide where similar declines have been documented following widespread adoption of fenestrated and branched endovascular techniques [22,23,24].

While endovascular techniques have changed TAAA management, several patient populations remain unsuitable for minimally invasive approaches [25]. In young patients, particularly those under 50 years of age, open repair was always preferred due to superior long-term durability and the need for lifelong aneurysm exclusion [6,25].

Another patient group in which open repair has traditionally been favoured is those with genetically triggered aortopathies. Early prospective experience from the various countries has challenged the traditional dogma that all patients with heritable aortopathy require open surgery [26]. This cohort of 171 genetically triggered aortopathy patients treated endovascularly demonstrated 98.8% technical success and 1.2% 30-day mortality, albeit at the cost of a 53.2% reintervention rate after 5 years [26]. Also smaller European series reporting mid-term durability when a synthetic landing zone is available, suggest that endovascular solutions can be acceptable in highly selective patients [27].

Also, the introduction of arch and iliac devices have become decisive “game-changers” for endovascular eligibility. The introduction of off-the-shelf branched-arch devices and custom fenestrated stent-grafts now permits secure proximal fixation in zones 0–2 without preceding frozen-elephant-trunk procedures [28]. However, anatomical constraints remain significant in patients with chronic dissections and true lumen collapse. This is reflected in our institutional experience, where post-dissection operations have increased since 2014. Although techniques such as STABILISE and membrane-slit show promise, they are associated with considerable complication rates—particularly in genetic aortopathy patients, where one-year anatomical outcomes are notably inferior compared to non-genetic cases [29].

Despite these endovascular advances, specific patient populations continue to require open intervention. Infection and aorto-enteric/aorto-bronchial fistulae remain absolute indications for open surgery. Systematic reviews demonstrate that aortoesophageal fistula carries an overall in hospital mortality rate of 42% [30,31]. While endovascular repair can serve as a bridging intervention for hemodynamically unstable patients, definitive management typically requires combined aortic graft replacement and esophagectomy to achieve respectable outcomes.

Some limitations need to be acknowledged. Selection bias is inherent in our observational design, as treatment decisions were based on surgeon preference and institutional protocols rather than randomized allocation. Other centers may have adopted endovascular techniques earlier than our institutions, potentially concentrating more complex cases unsuitable for endovascular repair at our centers during the transition period [32]. Furthermore, this observational study was conducted at two high-volume tertiary referral centers, introducing referral bias and limiting generalizability to lower-volume or differently resourced settings. Changes over time may introduce confounding in any long-running observational cohort; in this study, practice variability was limited by a largely unchanged operative protocol and standardized perioperative pathways across centers, yet temporal evolution in case-mix and supportive care cannot be excluded despite era stratification and adjusted analyses. Additionally, the retrospective design and concentration of cases may not reflect broader practice patterns. Missing data in primary endpoints and included covariates was low, and models were fit using complete-case analysis, which may still introduce minimal bias if missingness is not completely at random. As this was not a prospective multicenter study, our findings should be interpreted as descriptive of high-volume center experience rather than prescriptive for universal TAAA management. Long-term outcomes were not analyzed because the cohort spans two different eras with substantially unequal follow-up windows and evolving referral pathways limiting comparability

The preservation of open repair expertise remains crucial despite declining volumes. High-volume centers demonstrate superior outcomes not only due to surgeon experience but also through multidisciplinary team expertise, specialized protocols, and institutional infrastructure [33]. The centralization of complex aortic cases raises important questions about training and competency maintenance. Brazilian public health system data analyzing 556 procedures over 12 years demonstrated that hospitals performing higher volumes of open repairs achieved significantly better outcomes, with mortality rates inversely correlated with institutional experience [34]. The concentration of complex cases in fewer specialized centers may actually enhance outcomes through institutional learning curves, despite the overall reduction in case numbers [35]. Learning curve analyses for complex endovascular procedures demonstrate significant improvement in outcomes over time [28]. Evidence suggests that maintaining expertise in both open and endovascular techniques is essential for optimal patient care, as the two approaches should be considered complementary rather than competing modalities [36]. The ideal management strategy requires institutional capability in both techniques, allowing for individualized treatment selection based on patient-specific factors including age, anatomy, comorbidities, and life expectancy. This ensures that all patients receive the most appropriate intervention.

## 5. Conclusions

Despite the shift towards endovascular repair and the changing demographics of patients selected for open TAAA repair, specialized centers can maintain stable outcomes through standardized protocols and concentrated expertise. The preservation of open surgical capabilities remains crucial for specific patient populations, emphasizing the need for a balanced approach that integrates both open and endovascular techniques to provide optimal, individualized care.

## Figures and Tables

**Table 1:** Patient demographics comparing late-era and early-era.

		Overall (577)	Early Era (376)	Late Era (201)		
Variables	Categories	N (%)	N (%)	N (%)	*p*-Value	OR (95% CI)
Sex	Male	405 (69.50)	257 (68.35)	148 (71.64)	0.41	0.86 (0.58–1.24)
	Female	172 (29.81)	119 (31.65)	53 (26.37)	0.19	1.29 (0.88–1.90)
Age	Median (min-max)	60.75 (14–82.72)	63 (14–82.72)	55.87 (18.91–78)	<0.01	
BMI	Median (min-max)	25.17 (14.53–42.00)	25.18 (14.53–41.13)	25.15 (15.16–42.00)	0.98	
Genetically triggered aortopathy	Marfan	98 (16.98)	44 (11.70)	54 (26.87)	<0.01	0.36 (0.23–0.57)
	EDS	3 (0.52)	3 (0.80)	0 (0.00)	0.51	
	LDS	8 (1.39)	4 (1.06)	4 (1.99)	0.36	0.53 (0.12–2.37)
	Other	6 (1.04)	2 (0.53)	4 (1.99)	0.1	0.27 (0.03–1.50)
ASA-score	≥3	315 (54.59)	147 (39.10)	168 (83.58)	<0.01	0.13 (0.08–0.19)
	<3	59 (10.23)	36 (9.57)	23 (11.44)	0.48	0.82 (0.47–1.44)
Diabetes Mellitus		43 (7.45)	29 (7.71)	14 (6.97)	0.74	1.11 (0.58–2.22)
Renal insufficiency		239 (41.42)	163 (43.35)	76 (37.81)	0.17	1.28 (0.90–1.82)
Smoking		177 (30.68)	105 (27.93)	72 (35.82)	0.05	0.69 (0.48–1.00)
Hypertension		449 (77.82)	299 (79.52)	150 (74.63)	0.18	1.32 (0.88–1.98)
Heart failure		89 (0.15)	42 (11.17)	47 (23.38)	<0.01	0.42 (0.26–0.65)
Coronary artery disease		126 (21.84)	78 (20.74)	48 (23.88)	0.39	0.83 (0.55–1.26)
COPD		119 (20.62)	77 (20.48)	42 (20.90)	0.91	0.97 (0.64–1.50)
Myocardial infarction		150 (26.00)	133 (35.37)	17 (8.46)	<0.01	5.87 (3.50–10.41)
Previous aortic surgery		282 (48.87)	163 (43.35)	119 (59.20)	<0.01	0.53 (0.37–0.75)
Aortic dissection		292 (50.61)	162 (43.09)	130 (64.68)	<0.01	0.41 (0.29–0.59)
PCI		100 (17.33)	59 (15.69)	41 (20.40)	0.15	0.73 (0.47–1.14)
CABG		41 (7.11)	29 (7.71)	12 (5.97)	0.44	1.31 (0.66–2.73)

Age and BMI comparisons were conducted via Mann–Whitney U test or Chi^2^ test. ASA: American Society of Anesthesiologists; BMI: body mass index; CABG: coronary artery bypass grafting; COPD: chronic obstructive pulmonary disease; PCI: percutaneous coronary intervention; EDS: Ehlers Danlos Syndrome; LDS: Loeys–Dietz Syndrome.

**Table 2:** Intraoperative outcome comparing late-era and early-era.

		Overall (577)	Early Era (376)	Late Era (201)		
Variables	Categories	N (%)	N (%)	N (%)	*p*-Value	OR (95% CI)
Urgent or emergency repair	Elective	475 (82.32)	304 (80.85)	171 (85.07)	0.21	0.74 (0.46–1.17)
	Emergency	49 (8.49)	34 (9.04)	15 (7.46)	0.52	1.23 (0.66–2.38)
	Urgent	53 (9.19)	38 (10.11)	15 (7.46)	0.29	1.38 (0.75–2.67)
Crawford classification	I	171 (29.64)	109 (28.99)	62 (30.85)	0.64	0.91 (0.63–1.33)
	II	160 (27.73)	108 (28.72)	52 (25.87)	0.47	1.15 (0.79–1.71)
	III	124 (21.49)	83 (22.07)	41 (20.40)	0.64	1.10 (0.73–1.69)
	IV	93 (16.12)	62 (16.49)	31 (15.42)	0.74	1.08 (0.68–1.75)
	V	29 (5.03)	14 (3.72)	15 (7.46)	0.05	0.48 (0.22–1.03)
Massive transfusion		281 (48.70)	205 (54.52)	76 (37.81)	<0.01	1.97 (1.39–2.80)
Intraoperative splenectomy		66 (11.44)	56 (14.89)	10 (4.98)	<0.01	3.30 (1.71–7.04)
Intraoperative Heart rhythm disorder		26 (4.51)	16 (4.26)	10 (4.98)	0.69	0.84 (0.38–1.98)
Surgery time (min)	Median (min-max)	381 (100–3559)	372.5 (112–3559)	397 (100–935)	0.07	
Duration of stay (Hospital)	Median (min-max)	24 (1–247)	23 (1–247)	25 (1–199)	0.57	
Duration of stay (ICU)	Median (min-max)	10 (0–213)	10 (0–213)	9 (0–164)	0.83	

**Table 3:** Postoperative outcome comparing late-era and early-era.

		Overall (577)	Early Era (376)	Late Era (201)		
Variables	Categories	N (%)	N (%)	N (%)	*p*-Value	OR (95% CI)
Wound complications		93 (16.12)	65 (17.29)	28 (13.93)	0.30	1.29 (0.80–2.11)
Pulmonary complications		341 (59.10)	222 (59.04)	121 (60.20)	0.79	0.95 (0.67–1.35)
	Pneumonia	248 (42.98)	160 (42.55)	88 (43.78)	0.78	0.95 (0.67–1.35)
	ARDS	51 (8.84)	19 (5.05)	32 (15.92)	<0.01	0.28 (0.15–0.51)
Cardiac complications	Any	184 (31.98)	125 (33.24)	59 (29.35)	0.34	1.20 (0.83–1.74)
	Myocardial infarction	18 (3.12)	16 (4.26)	2 (1.00)	0.03	4.14 (1.15–28.64)
	AF	84 (14.56)	60 (15.96)	24 (11.94)	0.19	1.39 (0.85–2.36)
AKI	1	112 (19.41)	79 (21.01)	33 (16.42)	0.18	1.35 (0.87–2.14)
	2	122 (21.14)	65 (17.29)	57 (28.36)	<0.01	0.53 (0.35–0.80)
	3	8 (1.39)	2 (0.53)	6 (2.99)	0.02	0.18 (0.02–0.83)
Neurological complications	Any	171 (29.64)	105 (27.93)	61 (30.35)	0.54	0.89 (0.61–1.30)
	SCI	41 (7.11)	30 (7.98)	11 (5.47)	0.26	1.48 (0.74–3.18)
	Stroke	34 (5.89)	21 (5.59)	13 (6.47)	0.67	0.85 (0.42–1.79)
Bleeding complications		203 (35.18)	124 (32.98)	79 (39.30)	0.13	0.76 (0.53–1.09)
Sepsis		152 (26.34)	91 (24.20)	61 (30.35)	0.11	0.73 (0.50–1.08)
Operative Mortality		119 (20.62)	82 (21.81)	37 (18.41)	0.34	1.23 (0.80–1.92)

AKI: acute kidney injury; ARDS: acute respiratory distress syndrome; SCI: spinal cord ischemia.

## Data Availability

Data are available on request.

## Supplementary Materials

The following supporting information can be downloaded at: https://www.mdpi.com/article/10.3390/jcm14197088/s1, Table S1: Patient demographics per Crawford extent; Table S2: Perioperative Outcome per Crawford extent; Table S3: Postoperative outcome per Crawford extent; Table S4: Logistic regression both eras; Table S5: Logistic regression early era; Table S6: Logistic regression late era.
