# Supplementary material for: Shift in Patient Demographics of Open Thoracoabdominal Aortic Aneurysm Repair Patients in the Endovascular Era
Source: J Clin Med. 2025 Oct 8;14(19):7088. doi: 10.3390/jcm14197088 (PMC12525119; doi:10.3390/jcm14197088)
Supplement: Supplementary file 1 [file jcm-14-07088-s001.zip › jcm-3884982-supplementary.pdf]

Supplementary Table S1. Patient demographics per Crawford extent

		Early Era (pre 31-12-2013)					Late Era (After 01-01-2014)				
		Crawford Extent									
Variables	***Categories***	1	2	3	4	5	1	2	3	4	5
**Sex**	Male	66	74	55	54	8	42	38	28	24	12
**Age**	Median (min-max)	62 (14-80)	60.58 (19-82.18)	64 (29-78)	66 (23-82.72)	61.67 (44.13-77)	55.65 (18.91-75)	54.87 (22.55-71.66)	58 (25.25-78)	58.22 (35.13 -78)	49.31 (19.68-67.27)
**BMI**	Median (min-max)	25.45 (16.26-39.78)	24.76 (14.53-41.12)	24.80 (15.94-37.76)	26.18 (15.55-37.03)	21.55 (17.58-27.77)	25.35 (15.94-42)	24.82 (15.16-37.03)	25.17 (18.94-34.85)	25.22 (16.90 - 33.08)	24.31 (15.78-37.5)
**Genetically triggered aortopathy**	Marfan	11	24	7	2	0	12	16	14	7	5
	EDS	0	1	1	0	1	0	0	0	0	0
	LDS	1	1	1	1	0	1	3	0	0	0
	Other	1	1	0	0	0	2	1	0	0	1
**ASA**	≥3	41	47	32	20	7	41	47	37	29	14
	<3	11	11	9	4	1	7	11	3	1	1
**Diabetes melitus**		4	11	7	7	14	5	3	3	0	3
**Renal insufficiency pre-op**		41	43	45	29	5	21	17	17	17	4
**Smoking**		25	30	29	15	6	24	16	10	15	7
**Hypertension**		88	81	70	50	10	51	40	32	20	7
**Heart failure**		6	11	10	15	0	13	10	7	12	5
**KHK**		18	17	22	19	2	13	13	6	14	2
**COPD**		13	23	23	11	7	12	12	7	5	6
**Myocardial infarction**		13	14	13	14	0	3	2	2	8	2
**Previous aortic surgery**		45	52	40	22	4	30	33	30	16	10
**Aortic dissection**		58	63	26	12	3	43	40	28	11	8
**PCI**		12	21	18	7	1	12	11	7	9	2
**CABG**		6	8	7	8	0	2	3	2	4	1

Supplementary Table S2. Perioperative Outcome per Crawford extent

		Early Era (pre 31-12-2013)					Late Era (After 01-01-2014)				
		Crawford Extent									
Variables	***Categories***	1	2	3	4	5	1	2	3	4	5
**Urgent or emergency repair**	*Elective*	93	90	72	40	9	53	44	38	24	12
	Emergency	9	8	4	12	1	4	6	0	3	2
	Urgent	7	10	7	10	4	5	2	3	4	1
**Crawford-classification**	*I*	109	X	X	X	X	62	X	X	X	X
	II	X	108	X	X	X	X	52	X	X	X
	III	X	X	83	X	X	X	X	41	X	X
	IV	X	X	X	62	X	X	X	X	31	X
	V	X	X	X	X	14	X	X	X	X	15
**Massive transfusion**		48	76	42	32	7	15	29	17	12	3
**Incidental splenectomy**		6	16	13	18	4	3	2	2	2	1
**Surgery time (min)**	*Median (min-max)*	315 (112-3559)	441 (188-996)	391 (170-664)	344 (147-705)	343 (179-513)	317.5 (100-717)	473 (280-935)	420 (214-690)	414 (274-653)	351 (266-600)
**Duration of stay (Hospital)**	*Median (min-max)*	23 (2-214)	26.5 (1-150)	28 (1-247)	18 (1-110)	21.5 (2-47)	21 (1-113)	32.5 (1-199)	25 (4-130)	23 (1-112)	21 (1-89)
**Duration of stay (IC)**	*Median (min-max)*	8.5 (1-213)	13.5 (0-149)	12 (1-107)	7 (0-58)	9 (2-22)	7 (1-112)	18 (0-164)	9 (2-67)	7 (1-115)	7 (0-77)

Supplementary Table S3. Postoperative outcome per Crawford extent

		Early Era (pre 31-12-2013)					Late Era (After 01-01-2014)				
		Crawford Extent									
Variables	***Categories***	1	2	3	4	5	1	2	3	4	5
**Wound complications**		18	17	19	8	3	5	11	8	2	2
**Pulmonary complications**		62	69	58	27	6	38	34	26	16	7
	Pneumonia	46	51	38	20	5	26	25	20	13	4
	ARDS	3	10	3	3	0	5	10	13	3	1
**Cardiac complications**	*Any*	37	37	28	21	3	22	17	12	6	2
	Myocardial infarction	5	4	3	6	0	1	1	0	0	0
	AF	18	19	15	7	1	11	7	3	2	1
**AKI**	*Stage 1*	7	6	15	5	4	6	8	3	7	3
	Stage 2	6	8	6	8	0	1	4	6	3	2
	Stage 3	2	12	7	9	1	7	19	12	7	2
**Neurological complications**	*any*	32	42	20	9	2	20	21	8	8	4
	SCI	6	16	6	2	0	2	6	0	1	2
	Stroke	4	15	1	1	0	3	5	3	2	0
**Vascular complications**		35	38	26	21	4	21	27	15	11	5
**Sepsis**		16	29	29	14	3	13	23	14	8	3
**Operative Mortality**		18	28	15	19	2	7	12	8	8	2

Supplementary Table S4. Logistic regression both eras.

Variable	Estimate	Std. Error	z value	P-value
**Age**	0.0062	0.002	2.497	**0.01**
**Genetically triggered aortopathy**	-0.279	0.718	-0.389	0.697
**ASA score**	0.5242	0.471	1.114	0.265
**Smoking**	0.2303	0.216	1.066	0.286
**Heart failure**	0.0002	0.354	0.008	0.994
**Myocardial infarction**	0.7009	0.4567	1.535	0.38
**Any type of aortic dissection**	0.913	0.509	1.794	0.07
**Intraoperative massive transfusion**	0.968	0.520	1.831	0.06
**Incidental splenectomy**	0.238	0.660	0.361	0.718
**ARDS postoperative**	1.411	0.5431	2.597	**0.01**
**AKI stage 2**	1.766	0.7959	2.218	**0.03**
**AKI stage 3**	1.722	0.721	2.385	**0.02**

Supplementary Table S5. Logistic regression early era

Variable	Estimate	Std. Error	z value	P-value
**Age**	0.0047	0.0031	1.500	0.13
**Genetically triggered aortopathy**	-1.551	186.6	-0.008	0.99
**ASA score**	0.9908	0.5833	1.699	0.09
**Smoking**	0.3002	0.2879	1.043	0.30
**Heart failure**	0.00087	0.486	0.019	0.99
**Myocardial infarction**	0.578	0.579	1.000	0.32
**Any type of aortic dissection**	0.759	0.650	1.167	0.24
**Intraoperative massive transfusion**	2.214	0.919	2.408	**0.02**
**Incidental splenectomy**	-0.613	0.856	-0.716	0.47
**ARDS postoperative**	0.781	1.028	0.760	0.44
**AKI stage 2**	1.745	0.925	1.885	0.06
**AKI stage 3**	0.8133	0.8956	0.908	0.36

Supplementary Table S6. Logistic regression late era

Variable	Estimate	Std. Error	z value	P-value
**Age**	0.1043	0.05661	1.843	0.07
**Genetically triggered aortopathy**	1.961	1.463	1.341	0.18
**ASA score**	-1.387	1.359	-1.021	0.30
**Smoking**	0.7261	0.4975	1.460	0.14
**Heart failure**	-0.1176	0.8511	-0.138	0.89
**Myocardial infarction**	-0.9286	1.749	-0.531	0.60
**Any type of aortic dissection**	0.9121	1.231	0.741	0.46
**Intraoperative massive transfusion**	-0.7621	1.184	-0.644	0.51
**Incidental splenectomy**	6.674	3.260	2.048	**0.04**
**ARDS postoperative**	2.336	1.019	2.293	**0.02**
**AKI stage 2**	19.23	2412	0.008	0.99
**AKI stage 3**	22.01	2412	0.009	0.99
